# Identification and Characterization of *Fusarium proliferatum*, a New Species of Fungi that Cause Fungal Keratitis

**DOI:** 10.1038/s41598-018-23255-z

**Published:** 2018-03-20

**Authors:** Shengtao Sun, Qixue Lui, Lei Han, Qiufei Ma, Siyu He, Xiaohua Li, Hongmin Zhang, Junjie Zhang, Xiaohui Liu, Liya Wang

**Affiliations:** 1 The First Affiliated Hospital of Zhengzhou University, Zhengzhou City, China; 2 Henan Eye Institute, Henan Eye Hospital and Henan Key Laboratory of Ophthalmology and Visual Science, Henan Provincial People’s Hospital, People’s Hospital of Zhengzhou University, People’s Hospital of Henan University, Zhengzhou City, China

## Abstract

*Fusarium proliferatum* (*F. proliferatum*) is known as a pathogen of corn and other crops, but its role in fungal keratitis has not been well investigated. Among 877 *Fusarium* isolates, we identified 155 (17.7%) stains as *F. proliferatum* according to their morphological features and partial DNA sequencing of translation elongation factor-$1\alpha$ (EF-$1\alpha$) in this study. *In vitro* antifungal susceptibility tests showed that the *F. proliferatum* strains were sensitive to natamycin and vorionazole but resistant to amphotericin B, fluconazol, ketoconazole and itaconazole. Most of the *F. proliferatum*-positive keratitis patients (44/155,28.4%) were aged 51–60 years old. The main cause of infection was injury by a plant (51/155, 32.9%). A combination of 1% amphotericin B and 3% ketoconazole cured 45.2% (14/31) and a combination of 0.5% natamycin and 0.5% voriconazole cured 59.1% (13/22) of *F. proliferatum*-positive patients. The date suggests that *F. proliferatum* identified through EF-1ɑ DNA sequencing is an important new species that causes fungal keratitis. Based on antifungal susceptibility, treatment with a combination of 0.5% natamycin and 0.5% voriconazole improves the therapeutic efficacy in *F. prolifertum*-positive patients.

## Introduction

Fungal keratitis causes serious damage to vision, and 15–27% of patients with fungal keratitis undergo surgical intervention, such as corneal transplantation, removal of eye contents and enucleation, or suffer vision deterioration resulted from drug treatment failure. Several species of *Fusarium* can cause this disease, and more than 50% of all fungal keratitis are caused by this genus^1^. Fungal keratitis can be caused by different species of the genus *Fusarium*, including the *Fusarium solani* species complex (FSSC), *Fusarium moniliforme* (*Fusarium verticilliodes, F. Verticilliodes*)*, Fusarium avenascus* (*F. avenascus*)*, Fusarium oxysporum* (*F. oxysporum*)*, Fusarium poae* (*F. poae*), and *Fusarium dimerum* (*F. dimerum*)^1^. A new *Fusarium* species, *F. proliferatum*, had been reported in 0.2% of 67 fungal keratitis cases that resulted from contaminated contact lens preservation solution in the United States, Europe and Southeast Asia in 2007^2,3^.

*F. proliferatum* (Matsush.) Nirenberg, formerly Gerlach & Nirenberg 1982, is a fungus with a worldwide distribution that has been associated with a variety of diseases in important economical plants, including corn and bananas^4,5^. It can cause a disseminated infection in immunocompromised patients^6–10^ and abscesses in the body where trauma is caused by a plant^11,12^. Although *F. proliferatum* was mentioned in 2007^3^ as an agent that can cause *Fusarium* keratitis when found in contact lens preservation solution, the clinical features and outcomes associated with *F. proliferatum*-infected keratitis, and its etiological characteristics have not been reported.

In this study, we identified *F. proliferatum* obtained from keratitis cases using partial DNA sequencing of translation elongation factor (EF)-1α. We then evaluate the *in vitro* susceptibility of *F. proliferatum* to antifungal agents and describe the clinical features and results of treatment in *F. proliferatum-*positive keratitis patients.

## Results

### Patients

16839 patients with keratitis who presented with a corneal ulcer with a diameter larger than 2 mm at the Microbiological Department of Henan Eye Institute and who underwent a microorganism test from January 1st, 2005 to December 31, 2016 were included. Among these patients, hyphae were found in the corneal scraping of 10343 cases. A total of 5321 strains of fungi and 2197 strains of *Fusarium spp*. were isolated from 5321 cases (Table 1).

**Table 1:** The results of fungal tests performed in the Eye Institute from 2005 to 2016.

Year	Number of patients	Hyphae-positive on corneal scraping (n/%)	Fungal culture -positive (n/%)	*Fusarium spp*. (n/%)
2005	594	320/53.9	103/17.3	63/61.2
2006	732	434/59.3	283/38.7	182/64.3
2007	981	618/63.0	379/38.6	195/51.4
2008	1152	735/63.8	418/36.3	215/51.4
2009	1068	572/53.6	291/27.2	151/51.9
2010	1136	722/63.6	402/35.4	189/47.0
2011	1424	815/57.2	576/40.4	243/42.2
2012	1398	738/52.8	573/41.0	180/31.4
2013	1665	1024/61.5	585/35.1	262/44.8
2014	2488	1894/76.1	770/30.9	176/22.9
2015	2135	1308/61.3	483/22.6	153/31.7
2016	2066	1163/56.3	458/22.2	188/41.0
Total	16839	10343/61.4	5321/31.6	2197/41.3

### Identification of *F. proliferatum* in fungal keratitis

Among the 2197 strains of *Fusarium spp*. isolated from corneas in the Henan Eye Institute during 2005–2016, 891 strains were tested for the EF-1α gene using DNA sequencing. In all, 877 strains (98.4%) were positive for the EF-1α DNA sequence. Among these 877 strains, 533 (60.8%) were FSSC, 326 (37.2%) belonged to the *Gibberella fujikuroi* species complex (GFSC), 15 (1.7%) belonged to *Fusarium oxysporum* species complex (FOSC), and 3(0.3%) were *Fusarium spp*. There were 3 species in GFSC, including *F. proliferatum* (155 /877,17.7%), *F. verticillioides* (148 /877,16.9%) and GFSC (23 /877,2.6%) (Table 2).

**Table 2:** The genotypes and species of *Fusarium* strains obtained from patients with fungal keratitis and tested against the EF-1α DNA sequence.

Gene type	n	Percentage	Gene type	n	Percentage
FSSC	2	0.23	FSSC6-f	12	1.37
FSSC 1-a	2	0.23	FSSC6-g	3	0.34
FSSC 2-b	10	1.14	FSSC6-h	16	1.82
FSSC 2-e	6	0.68	FSSC6-j	3	0.34
FSSC 2-k	18	2.05	FSSC8-b	1	0.11
FSSC 2-p	1	0.11	FSSC 11-a	1	0.11
FSSC 2p-a	1	0.11	FSSC 11-c	1	0.11
FSSC 3 + 4-a	2	0.23	FSSC 12-e	1	0.11
FSSC 3 + 4-aa	2	0.23	FSSC 14-c	1	0.11
FSSC 3 + 4-e	2	0.23	FSSC 16-a	1	0.11
FSSC 3 + 4-eee	85	9.69	FSSC 18-a	4	0.46
FSSC 3 + 4-ii	56	6.39	FSSC 18-b	1	0.11
FSSC3 + 4-jj	2	0.23	FSSC 24-a	2	0.23
FSSC 3 + 4-pp	9	1.03	FSSC 29-a	3	0.34
FSSC 3 + 4-ss	14	1.60	FSSC 34-a	4	0.46
FSSC 3 + 4-z	45	5.13	FSSCclade2	2	0.23
FSSC 5–1	3	0.34	*F. proliferatum*	155	17.67
FSSC 5-a	1	0.11	*F. verticillioides*	148	16.88
FSSC 5-b	5	0.57	GFSC	23	2.62
FSSC 5-c	1	0.11	FOSC	3	0.34
FSSC 5-d	168	19.16	FOSC16	3	0.34
FSSC 5-g	19	2.17	FOSC22	1	0.11
FSSC 5-h	8	0.91	FOSC203	1	0.11
FSSC 5-j	1	0.11	FOSC232	1	0.11
FSSC 5-k	1	0.11	FOSC67	2	0.23
FSSC 6-a	6	0.68	FOSC197	4	0.46
FSSC 6-b	1	0.11	*Fusarium spp*.	3	0.34
FSSC6-d	1	0.11	Total	877	100
FSSC 6-e	5	0.57			

FSSC indicates *Fusarium solani* species complex; GFSC indicates *Gibberella fujikuroi* species complex; and FOSC is the *Fusarium oxysporum* species complex.

**Figure 1:**
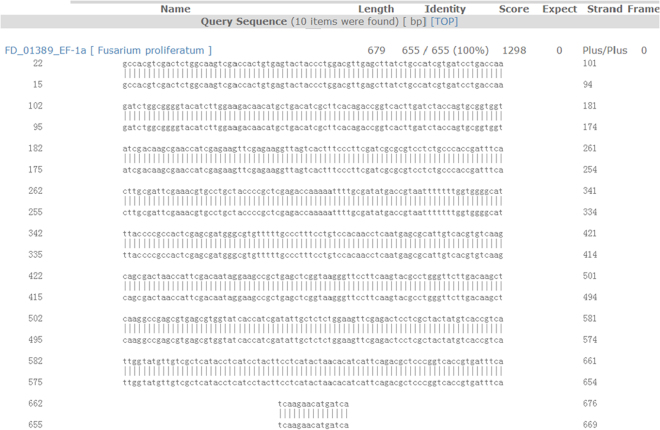
The BLAST results of EF-1α DNA sequencing of strain 80458 with the FD_01389-EF-1α sequence of *F. proliferatum* in *Fusarium* ID v 1.0. The EF-1α DNA sequence of strain 80458 was 693 bp long. It fully matched the FD_01389-EF-1α sequence from base 22 to base 676 and the FD_01389-EF-1α sequence from base 15 to base 669 with an identity of 100%. The total matched length was 655 bp.

The length of the DNA sequence of EF-1α that was used to test for *F. proliferatum* strains was 633 bp to 693 bp long. The EF-1α DNA sequence of strain 80458 was 655 bp long and was a complete match for FD_01389 of *F. proliferatum* in *Fusarium* ID v 1.0 (The Basic Local Alignment Search Tool (BLAST) result shown in Fig. 1). Bases 22 to 676 of strain 80458 completely matched bases 15 to 669 of FD_01389 (identity, 100%).

The EF-1α DNA sequence of 138 of the tested *F. proliferatum* strains matched FD_01389 of *F. proliferatum* in *Fusarium* ID v 1.0, and 19 of the tested *F. proliferatum* strains matched FD_01380 of *F. proliferatum* in *Fusarium* ID v 1.0. The lengths of the matched EF-1α DNA sequences ranged from 607 bp to 667 bp, and identity ranged from 96.2% to 100%. Among the 155 EF-1α DNA sequences that were matched in the tested strains, 5 (3.2%) matched with an identity of 100%, 130 (83.9%) matched with an identity of more than 99% but less than 100%, 16 (10.3%) matched with an identity of more than 98% but less than 99%, 2 (1.3%) matched with an identity of more than 97% but less than 98%, and 2 (1.3%) matched with an identity of more than 96% but less than 97%.

### Morphological features of *F. proliferatum*

**Figure 2:**
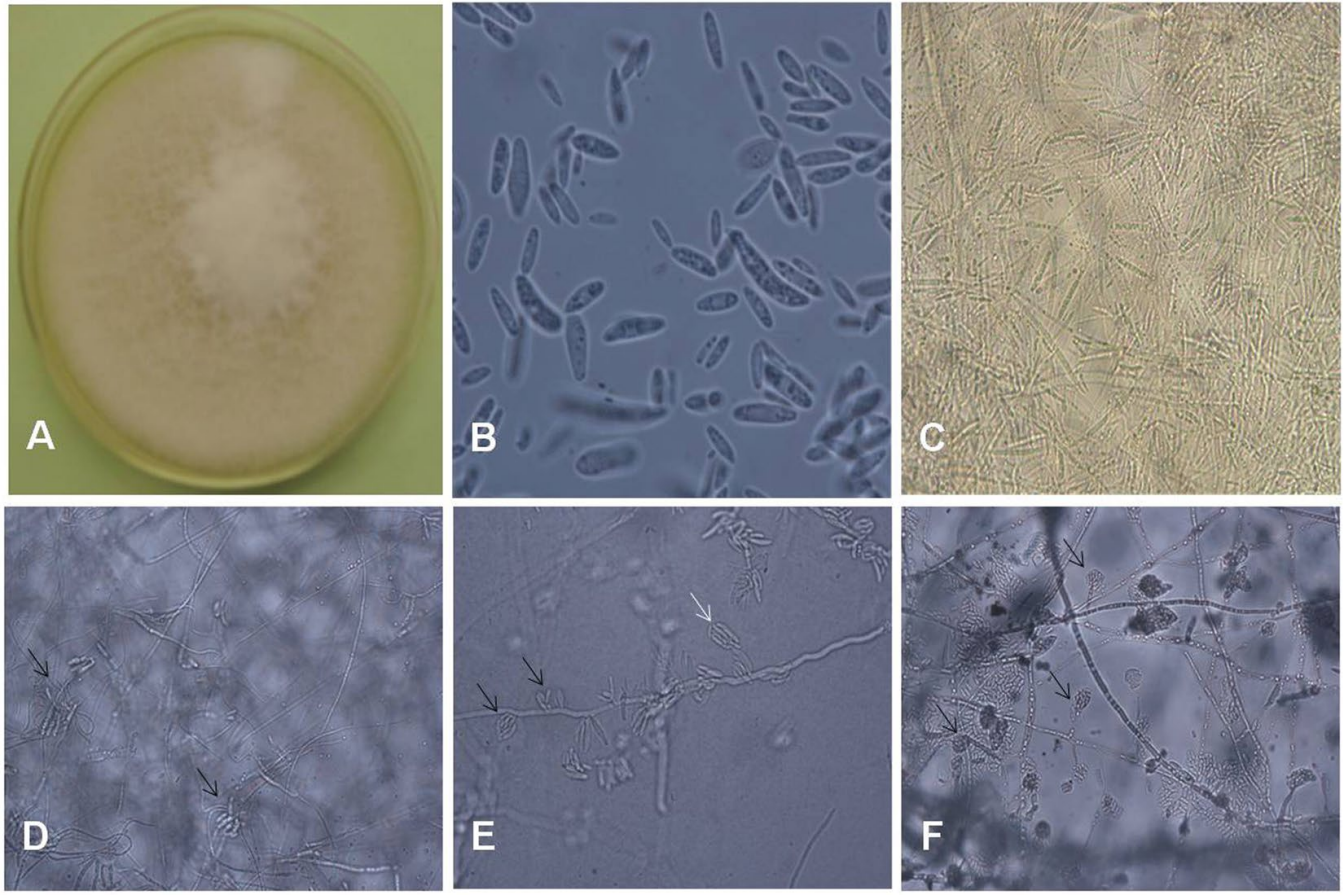
(**A**) *F. proliferatum* appears as white villous colonies that produce light purple pigment and have a diameter of 7 mm after 7 d of growth on PDA at 27 °C. (**B**) There were a large number of slender microconidia with no septa that were 2 × 10–15 µm in size, as shown on a 10% KOH wet film of PDA after 7 d (×1000). (**C**) A large number of slender sickle-shaped macroconidia with tips at two ends were observed on the 10% KOH wet film of PDA after 7 d. The macroconidia with 1 septum were 2 × 26 µm, and the macroconidia with 3 septa were 2 × 48 µm (×400). (**D**) A large number of sickle-shaped macroconidia with one to three septa that had slender mycelia connected to hyphae at both ends (indicated by black arrows) were observed on 10% KOH wet film on PDA after 7 d. Macroconidia were present in groups (×400). (**E**) A large number of false heads that contained 8–16 robust sickle-shaped conidia with no septa on both monophialides (indicated by white arrow) and polyphialides (indicated by black arrows), as shown on 10% KOH wet film of PDA after 7 d. They were 4.5 × 6–15 µm in size (×400). (**F**) A large number of false heads containing microconidia on monophialides are shown on PDA after 3 d. They were 2 × 6 µm in size (×400).

We reexamined the morphology of the isolated *F. proliferatum* specimens after we obtained the EF-1α DNA sequence data. We found that *F. proliferatum* had some distinguishing morphological features. *F. proliferatum* produced white villous colonies with a diameter of 7 mm and produced light purple pigment after 7 d on potato dextrose agar (PDA) at 27 °C (Fig. 2A). A large number of slender microconidia with no septa were 2 × 10–15 µm in size (Fig. 2B), a large number of slender sickle macroconidia (2 × 26 µm) had one septum, some slender sickle macroconidia (2 × 48 µm) had 3 septa with tips at two ends (Fig. 2C), a large number of sickle macroconidia had one to three septa with slender mycelium that were connected to hypha at both ends (Fig. 2D), and a large number of false heads containing 8–16 robust sickle conidia that had no septa were present on both monophialides and polyphialides (Fig. 2E) in 10% KOH wet films grown on PDA for 7 d. Microcultures grown on PDA for 3 d produced a large number of false heads that contained microconidia (Fig. 2F) on monophialides.

### Epidemiological characteristics of *F. proliferatum*-positive patients

Of the 155 patients who were *F. proliferatum* positive, 97 were male and 58 were female. The right eye was infected in 83 patients, and the left eye was infected in 72 patients. The patients’ ages ranged from 7 to 83 years old, and their average age was 51.4 ± 13.7 years old. The group with the most patients ranged in age from 51–60 years old (44/155, 28.4%) and was followed by the group containing 41–50-year-old patients (36/155, 23.2%) (Table 3).

**Table 3:** The distribution of the ages and duration of symptoms of the 155 *F. proliferatum* -positive patients in the eye hospital.

Age (years old)	Cases	Percentage	Duration (days)	Cases	Percentage
0–10	1	0.6	0–10	40	25.8
11–20	2	1.3	11–20	69	44.5
21–30	8	5.2	21–30	28	18.1
31–40	25	16.1	31–60	13	8.4
41–50	36	23.2	>60	5	3.2
51–60	44	28.4			
61–70	31	20.0			
>70	8	5.2			

The patients who presented to our eye hospital complained of eye redness, streaming eyes, eye irritation, photophobia and hypopsia. The patients presented from the third to the 720th day after symptom onset, and the average duration of their symptoms was 25.4 ± 58.8 days. In all, 137 (88.4%) patients presented to our eye hospital before the 30th day after symptom onset (Table 3). *F. proliferatum* was most often observed in October (42/155, 27.1%) and November (33/155, 21.3%). In this study, 91.0% (144/155) of the *F. proliferatum*-positive patients originated from Henan Province, and the other patients were from neighboring provinces, including Anhui Province, Hebei Province and Shanxi Province, in China. In all, 40.0% (62/155) of the *F. proliferatum*-positive patients had no history of trauma, and 32.9% (51/155) of the patients were injured by a plant (Table 4).

**Table 4:** The events that caused 155 cases of *F. proliferatum* keratitis in the eye hospital.

Vulnerant		Number	percentage
Plants	Corn^a^	30	19.4
	Other crops^b^	11	7.1
	Branches	8	5.2
	Grass	2	1.3
Animal^c^		6	3.9
Non-living materials^d^		18	11.6
Chemical damage		1	0.6
Secondary infection of malnutrition		2	1.3
Stay up late		1	0.6
After agricultural labor		5	3.2
Unidentified damage		9	5.8
No history of trauma		62	40.0

^a^Includes corn leaves, seeds and stem; ^b^includes rice, cotton, beans, peanut and sorghum; ^c^includes flying insects and eyelashes; ^d^includes dust, powder, barber knife, iron scurf *et al*.

### Clinical features of *F. proliferatum-*positive patients

**Figure 3:**
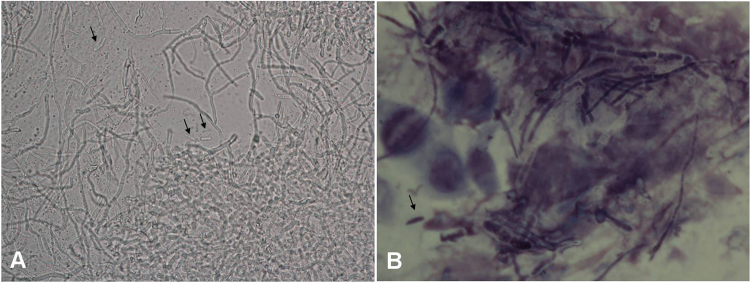
Hyphae morphology of *F. proliferatum* in corneal scrapings. (**A**) A large number of hyphae with a diameter of approximately 2 µm and vertical branches were obtained from case 90853 and are shown on a 10% KOH wet film of a corneal scraping under microscopy. Stick-shaped conidia (indicated by black arrows) are shown (×400). (**B**) A large number of purple fungal diaphragm-hyphae with a diameter of approximately 2 µm were found in the corneal scraping obtained from Case 100862. The scraping was stained with Giemsa stain and observed under a microscope. A sickle-shaped conidia (indicated by a black arrow) was found (×1000).

The corneal lesions caused by *F. proliferatum* were white and gray, and most of the ulcers were on the central cornea. The diameters of the corneal ulcers ranged from 2 mm to the size of the cornea. In 27 patients (17.4%), the ulcer was larger than 7 mm. Hypopyon was documented in 20 patients (12.9%). Hyphae were found in the corneal scrapings obtained from 151 patients (97.4%) and could be detected as early as 3 days post-infection. We reviewed the corneal smears made from tissues obtained from 76 *F. proliferatum-*positive patients. A large number of long hyphae with diameters of approximately 2 µm and vertical angle branches were observed in the corneal scrapings obtained from 48 patients (48/76, 63.2%) (Fig. 3A). Stick-shaped conidia were observed in the corneal scrapings obtained from 6 cases (6/76, 7. 9%) (Fig. 3B).

### *In vitro* antifungal susceptibility to *F. proliferatum*

Bacteriostatic rings were made using 31 strains of *F. proliferatum* to test their susceptibility to antifungal drugs from 2013 to 2016 (Table 5). The results showed that while *F. proliferatum* is sensitive to natamycin and vorionazole, all of the *F. proliferatum* samples were resistant to terbinafine, amphotericin B, fluconazol, ketoconazole and itraconazole.

**Table 5:** *In vitro* antifungal susceptibility in 31 strains of *F. proliferatum* obtained from 2013 to 2016.

Drugs	Number	S (n/%)	R (n/%)	χ ± SD (mm)	Range (mm)
Natamycin	29	28/96.6	1/3.4	29.6 ± 5.3	8–38
Vorionazole	29	28/96.6	1/3.4	28.7 ± 7.0	8–46
Terbinafine	29	11/37.9	18/62.1	20.0 ± 6.8	12–40
Amphotericin	20	1/5.0	19/95.0	10.8 ± 2.9	9–20
ketoconazole	20	0	20/100	10.0 ± 1.4	8–12
Itaconazole	19	0	19/100	10.3 ± 2.6	8–18
Fluconazole	28	0	28/100	9.4 ± 1.2	8–14

S, sensitive; R, resistant.

### Therapeutic outcomes in *F. proliferatum-*positive patients

We followed up 53 patients, including 31 patients who were followed in 2005–2012. Fourteen (14/31, 45.2%) *F. proliferatum*-positive patients were cured by applying drops of 1% amphotericin B and 3% ketoconazole. The duration of drug treatment was from 5 weeks to 5 months. Seventeen (17/31, 54.8%) were cured by surgery. Five patients underwent lamellar keratoplasty (LK), 10 patients underwent penetrating keratoplasty (PK), 1 patient underwent enucleation of the eye combined with hydroxyapatite ocular prosthesis implantation after PK failure, and 1 case underwent enucleation of the eye combined with hydroxyapatite ocular prosthesis implantation because the drops treatment failed.

In all, 22 patients were followed up in 2013 and 2016. The antifungal susceptibility tests showed that most of the *F. proliferatum* isolates were sensitive to natamycin and vorionazole. The patients were therefore treated using a combination of 0.5% natamycin and 0.5% voriconazole. Among these 22 patients, 13 cases (59.1%) were cured by the drops. The duration of drug treatment ranged from 4 weeks to 5 months. Surgeries were performed in 9 patients, including 2 LK, 3 PK and 4 enucleations.

A comparison of the results related to the duration of symptoms and the diameter of corneal ulcers between the drug treatment group and the operation group in these 53 patients showed that the duration of symptoms in the operation group (20.1 d) was longer than that in the drug group (16.5 d) (*p* = 0.010) and that the diameters of the corneal ulcers were larger in the operation group (7.0 mm) than in the drug group (5.3 mm) (*p* = 0.000). However, there was no significant difference in the ages of the patients between the operation group (50.5 years old) and the drug group (51.2 years old) (*p* = 0.645). A comparison of the results showed that surgery should be performed in patients with a corneal ulcer larger than 7 mm.

### The features of case 939938, concerning a patient who was *F. proliferatum-*positive

**Figure 4:**
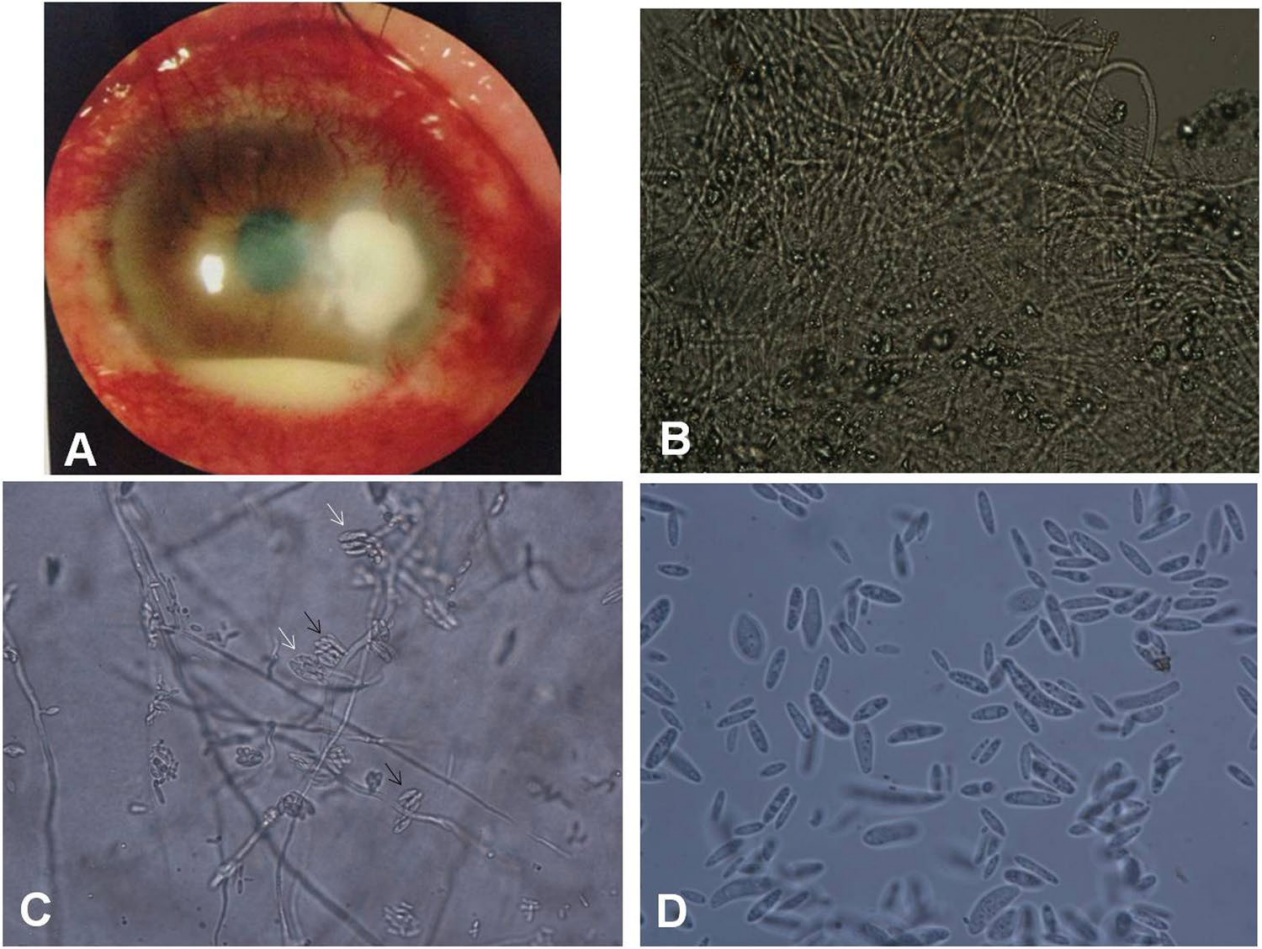
The features of case 939938, which was *F. proliferatum*-positive. (**A**) Under a slit-lamp microscope, the left eye exhibited severe conjunctival injection and a next-to-central corneal ulcer (5 × 5 mm) with white, thick, irregular necrosis and irregular edges. A hypopyon of 3 mm in height was observed (×10). (**B**) A large number of fungal hyphae with a diameter of approximately 2 µm were observed under a microscope in corneal scrapings on 10% KOH wet film (×400). (**C**) A large number of false heads containing robust sickle-shaped conidia with no septa were present on both monophialides (indicated by white arrows) and polyphialides (indicated by black arrows), as shown on 10% KOH wet films of PDA after 7 d. The conidia were 4.5 × 6–15 µm in size (×400). (**D**) A large number of slender microconidia with no septa, sizes of 2 × 10–15 µm and some robust sickle or rod-shaped conidia (indicated by black arrows) were observed on a 10% KOH wet film of PDA after 7 d (×1000).

A 63-year-old man from Sanmenxia City, Henan Province, presented to our institute on April 5, 2011 complaining of left eye pain for 4 days in addition to photophobia, tears, and decreased vision. The left eye exhibited severe conjunctival injection and a central corneal ulcer (5 × 5 mm) that displayed white, thick, irregular necrosis and irregular edges when examined using a slit-lamp microscope (Fig. 4A). A hypopyon of 3 mm in height was observed. A large number of fungal hyphae with diameters of approximately 2 µm were observed using microscopy in corneal scrapings on 10% KOH wet film (Fig. 4B). A purple colony grew on PDA medium after 7 d of inoculation at 27 °C. A large number of false heads containing robust sickle conidia with no septa of 4.5 × 6–15 µm in size were observed on both monophialides and polyphialides on 10% KOH wet films grown on PDA for 7 d (Fig. 4C). We also observed a large number of slender microconidia of 2 × 10–15 µm in size and containing no septa but with some robust sickle or rod conidia (Fig. 4D). These fungi were identified as *F. proliferatum* based on their morphology. Sequencing of EF-1α DNA showed that the samples contained a 681 bp DNA fragment of this gene. BLAST results showed that this sequence matched FD_01389 of *F. proliferatum* in *Fusarium* ID v 1.0 with an identity of 99.84%. The patient was initially administered eye drops containing 1% amphotericin B and 3% ketoconazole, but the disease progressed. The patient was cured by PK after 8 days in the hospital and had not experienced recurrence after 2 years.

## Discussion

FSSC is the most common pathogen that causes fungal keratitis. However, the role of *F. proliferatum* in this disease has not been well investigated. However, we found that *F. proliferatum* is an important *Fusarium* species in fungal keratitis in China, where it was isolated from 877 cases of *Fusarium* keratitis at a rate of 17.7%. We describe more than one hundred cases of *F. proliferatum* keratitis, and provide a detailed description of the identification of this species and its epidemiological and clinical features. Because it can cause serious visual damage, clinicians should pay more attention to it.

*F. proliferatum* is one species in the *Fusarium* genus. It is in the *Liseola* group with *F. verticilliodes*, *F. subglutinans*, and *F. anthophilum*. According to Nelson PE, the above species are distinguished by their morphology, the mode of formation of their microconidia and the morphology of their microconidiophores. *F. proliferatum* has the same characteristics as *F. verticilliodes* except that its microconidia are borne in short chains and possess false heads on both monophialides and polyphialides^13^.However, these subtle differences seem to have little effect on the identification of *Fusarium* species in a clinical laboratory setting.

Many studies have explored the usefulness of the EF-1α gene^14–17^, the ribosomal DNA internal transcribed spacer (ITS)^18,19^, and the β-tubulin gene^17,20^ for identifying *Fusarium* species. Partial sequencing of the EF-1α gene in *Fusarium* has been found to accurately and rapidly identify *Fusarium* species. As an essential component of the protein translation machinery, EF-1α is highly informative at the species level in *Fusarium*. Non-orthologous copies of the gene have not been detected in the genus, and universal primers have been designed that work across the phylogenetic breadth of the genus^15^. In this study, 98.4% of the *Fusarium* identified according to morphological features were also positive for EF-1α. The morphological review showed that the results of testing for *F. proliferatum* using EF-1α sequences were in agreement with the results obtained when morphological characteristics were used to identify the species. The level of identity of the EF-1α sequences was higher than 96% in all *F. proliferatum* tested in this study, and in 5 cases, it was 100%. These results showed that while morphological features can be used to identify *Fusarium*, the EF-1α DNA sequence of *F. proliferatum* is species-specific. We therefore suggest that a molecular method should be used to identify *Fusarium* species in a clinical setting.

*F. proliferatum* is a serious pathogen in fungal keratitis. Approximately 20% of affected patients have a large corneal ulcer (a diameter larger than 7 mm), and half of all *F. proliferatum*-positive patients will require an operation, with enucleation being performed in 11.3% of these patients (6/53). The epidemiological results showed that the longer the illness, the greater the chance that it will require surgical treatment. Therefore, it is crucial to obtain a rapid diagnosis and immediately initiate treatment in this condition. Hyphae are observed in corneal smears in more than 95% of affected patients and can be detected on corneal scraping as early as 3 days post infection. Obtaining corneal scrapings is essential to determining what treatment to use and should be performed soon as a corneal ulcer is found. We also found that the hyphae of *F. proliferatum* display unique morphological characteristics, including a 2 µm diameter with a vertical offset, and this can help determine which fungal species is present so that a proper prognosis can be achieved.

The *in vitro* susceptibility test was useful for selecting which antifungal drugs to use. The results of *in vitro* susceptibility tests showed that all of the tested strains of *F. proliferatum* were sensitive to natamycin and voriconazole. More *F. proliferatum*-positive patients were cured by a combination of 0.5% natamycin and 0.5% voriconazole (59.1%) than a combination of 1% amphotericin B and 3% ketoconazole (45.2%). These results suggest that the combination of 0.5% natamycin and 0.5% voriconazole has therapeutic efficacy in *F. prolifertum*-positive keratitis patients.

Following molecular identification, we observed slight differences in the morphology, EF-1α DNA sequence and fungal susceptibility among different strains of *F. proliferatu*m. If a fungal strain was identified as *F. proliferatum* using the EF-1α sequence and morphology, then voriconazole and natamycin were used to treat the fungal keratitis patient. This strategy will be beneficial for the treatment and prognosis of the keratitis caused by *F. proliferatum*.

## Methods

### Fungal test and isolation procedures

This study was approved by the Institutional Review Board of Henan Eye Institute. All experiments and methods of this study were performed in accordance with the international code of ethics on human biomedical research and the Helsinki declaration and an informed consent was obtained from every patient in this study. On presentation, corneal specimens were obtained from patients who were anesthetized using 5% tetracaine hydrochloride eye drops. The specimens were stained using 10% potassium hydroxide (KOH) and Giemsa staining, inoculated on Sabouraud’s dextrose agar (SDA), blood agar and brain–heart infusion broth to obtain fungal and bacterial cultures, and then evaluated for the presence of hypha and bacteria using microscopy.

### Fungal morphological identification

The fungal cultures were scored as positive if a fungal colony was found on SDA. *Aspergillus spp*. and *Penicillium spp*. were transferred to Czapek’s agar, whereas *Fusarium spp*., *Alternaria spp*. and other fungal genera were transferred to PDA for species identification according to morphological features. *Fusarium species* were identified according to their macroscopic characteristics, which included colony morphology, color, the growth rate of molds, the microscopic characteristics of their hyphae, spores and conidia, and the relationships among these characteristics in specimens grown on PDA. The isolated *Fusarium* strains were stored on PDA at 2–8 °C.

### Molecular identification of *Fusarium spp*

DNA sequencing of the EF-1α gene was performed in 891 *Fusarium* strains that were isolated from keratitis patients from 2005 to 2016 in our hospital.

Each fungal strain recovered on PDA at 26–28 °C for 1 week and was then inoculated on potato dextrose broth and grown at 26–28 °C for 4 days. The fungal mycelia were harvested by centrifuging the samples at 3000 g for 5 min, freezing them in liquid nitrogen and then crushing them to a fine powder. Genomic DNA was extracted from each culture using a Microbial DNA Extraction Kit (Shanghai Lifeng Biotechnology Co., Ltd., China) according to the instructions provided in the manual.

The primers used to sequence EF-1α and the PCR reaction conditions were previously described by O’Donnell^21,22^. The following primers (synthesized at Sangon Biotech (Shanghai, China) Co., Ltd.) were used to sequence EF-1α: EF1, 5′-ATG GGT AAG GAA GAC AAG AC-3′ and EF2, 5′- GGA AGT ACC AGT GAT CAT GTT-3′. The PCR reactions were performed in 50 µL reaction mixtures that contained 1 µL of each primer (25 µM), 2 µL of a DNA sample, 25 µL of PCR Master Mix (Fermentas Life Sciences, CA) and 21 µL of nuclease-free water. The samples were placed in an Applied Biosystems 9700 thermocycler (Emeryville, CA) and run using the following program: 1 cycle for 90 s at 94 °C, 40 cycles of 30 s at 94 °C, 30 s at 55 °C and 1 min at 72 °C, and 1 cycle for 5 min at 72 °C. The reactions were then held at 4 °C until collected. Positive PCR products were sequenced by Sangon Biotech (Shanghai) Co., Ltd.

The species and genotypes of *Fusarium* were identified through BLAST searches of the *Fusarium* ID v1.0 using the partial DNA sequences of EF-1α as the query.

### *In vitro* antifungal sensitivity test

Bacteriostatic rings of *Fusarium* strains were exposed to voriconazole, ketoconazole, terbinafine, natamycin, fluconazole, amphotericin B and itraconazole and tested using the agar diffusion method of the Clinical and Laboratory Standards Institute (CLSI) M-51^23,24^ during 2013 and 2016. The resistance breakpoint of the bacteriostatic rings to each drug was defined as <20 mm^24^.

### The patients treatment processes

The patients diagnosed with fungal keratitis in 2005–2012 were treated by antifungal therapy applying drops of 1% amphotericin B and 3% ketoconazole at 20-min intervals after hyphae were tested in the corneal scraping under microscopy. In 2013 and 2016, all patients were first treated with a combination of drops containing 0.5% natamycin, 0.5% voriconazole and 0.5% terbinafine, and then used sensitive drops based on antifungal susceptibility tests. If antifungal therapy failed to control the disease progress more than 20 days, surgeries were performed, including LK, PK, or enucleation. All patients were followed up at least 2 years, and they were evaluated as cured when the corneal ulcer was healing and the symptoms disappeared.

### Statistics analysis

An Excel table was constructed, and SPSS 16.0 software was used to analyze the data. Frequencies, means and Student’s *t* test were used to analyze epidemiological manifestations, clinical characteristics and treatment results in the patients. The percentages of resistance and sensitivity were calculated for all of the ocular isolates obtained during this study.
